# Supplementary material for: Xinghamide A, a New Cyclic Nonapeptide Found in Streptomyces xinghaiensis
Source: Mar Drugs. 2023 Sep 26;21(10):509. doi: 10.3390/md21100509 (PMC10608500; doi:10.3390/md21100509)
Supplement: Supplementary file 1 [file marinedrugs-21-00509-s001.zip › marinedrugs-2612931-supplementary.pdf]

Supporting Information for

Article

Xinghamide A, a New Cyclic Nonapeptide Found in *Streptomyces xinghaiensis*

Soohyun Um [1,†], Jaeyoun Lee [1,†], Sung Jin Kim [2], Kyung A Cho [1], Ki Sung Kang [2] and Seung Hyun Kim [1,*]

1 College of Pharmacy, Yonsei Institute of Pharmaceutical Sciences, Yonsei University, Incheon 21983, Republic of Korea; soohyunum@yonsei.ac.kr (S.U.); jaeyoun1024@yonsei.ac.kr (J.L.); kacjjang@gmail.com (K.A.C.)

2 College of Korean Medicine, Gachon University, Seongnam 13120, Republic of Korea; sungjinkim001@gmail.com (S.J.K.); kkang@gachon.ac.kr (K.S.K.)

* Correspondence: kimsh11@yonsei.ac.kr

† These authors contributed equally to this work.

Table of Contents

Figure S1. The bacterial strain *S. xinghaiensis* YSL1 cultivated on YPM agar plate after 14 days.

Figure S2. GNPS molecular networking of an ethyl acetate crude extract of liquid culture, an ethyl acetate crude extract of solid culture, and a methanol crude extract of solid culture after 14 days of cultivation of *S. xinghaiensis* YSL1.

Figure S3. ^{1}H NMR spectrum (850 MHz) of xinghamide A (**1**) in DMSO-d_6.

Figure S4. ^{13}C NMR spectrum (212.5 MHz) of xinghamide A (**1**) in DMSO-d_6.

Figure S5. HSQC NMR spectrum of xinghamide A (**1**) in DMSO-d_6.

Figure S6. COSY NMR spectrum of xinghamide A (**1**) in DMSO-d_6.

Figure S7. HMBC NMR spectrum of xinghamide A (**1**) in DMSO-d_6.

Figure S8. Magnified HMBC NMR spectrum (δ_C: 160-180 ppm; δ_H: 7.5-8.3 ppm) of xinghamide A (**1**) in DMSO-d_6.

Figure S9. TOCSY NMR spectrum of xinghamide A (**1**) in DMSO-d_6.

Figure S10. ROESY NMR spectrum of xinghamide A (**1**) in DMSO-d_6.

Figure S11. TIC of *S. xinghaiensis* YSL1 culture extract and EIC of xinghamide A (**1**).

Figure S12. Advanced Marfey's analysis of xinghamide A (**1**).

Figure S13. GITC analysis of hydrolysate of **1** to confirm configuration of β carbon of threonine in **1**.

Figure S14. HRESIMS/MS analysis of xinghamide A (**1**).

Table S1. LC/MS analysis of L, D-FDLA derivatives of the amino acids in xinghamide A (**1**).

Figure S1. The bacterial strain *S. xinghaiensis* YSL1 cultivated on YPM agar plate after 14 days.

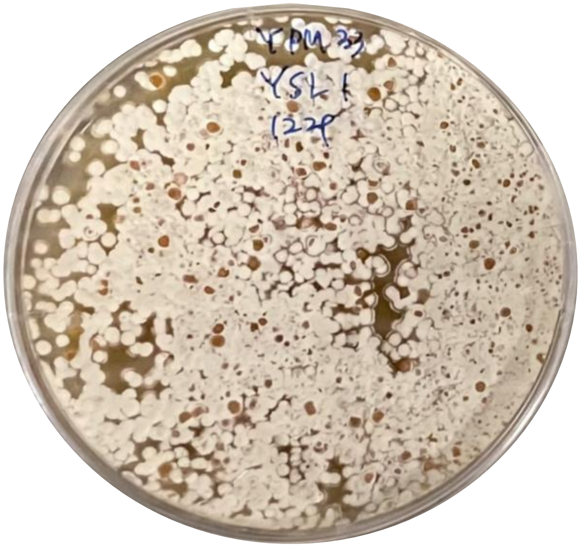

Figure S2. GNPS molecular networking of an ethyl acetate crude extract of liquid culture, an ethyl acetate crude extract of solid culture, and a methanol crude extract of solid culture after 14 days of cultivation of *S. xinghaiensis* YSL1.

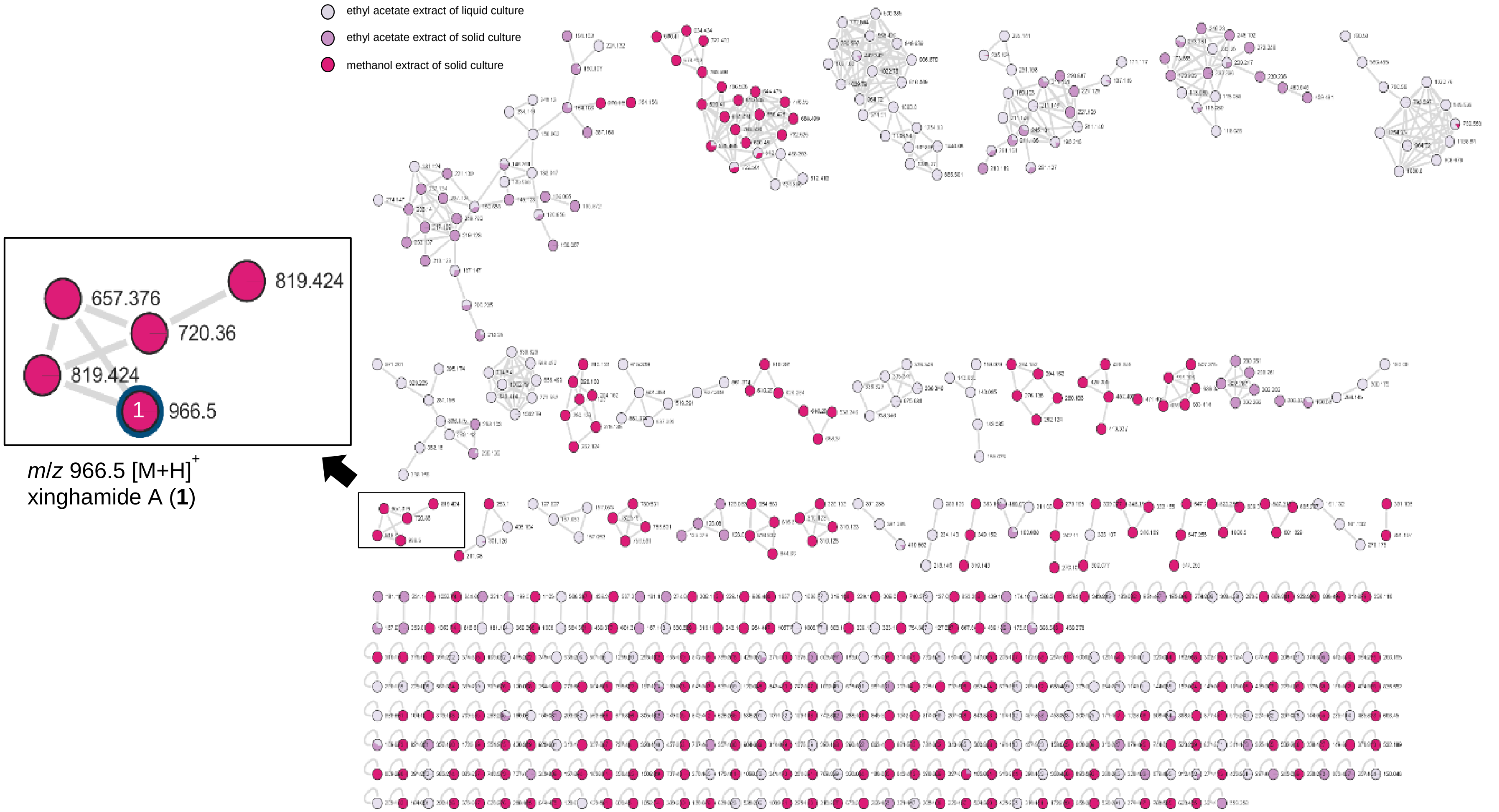

Figure S3. ^{1}H NMR spectrum (850 MHz) of xinghamide A (**1**) in DMSO-*d*$_6$.

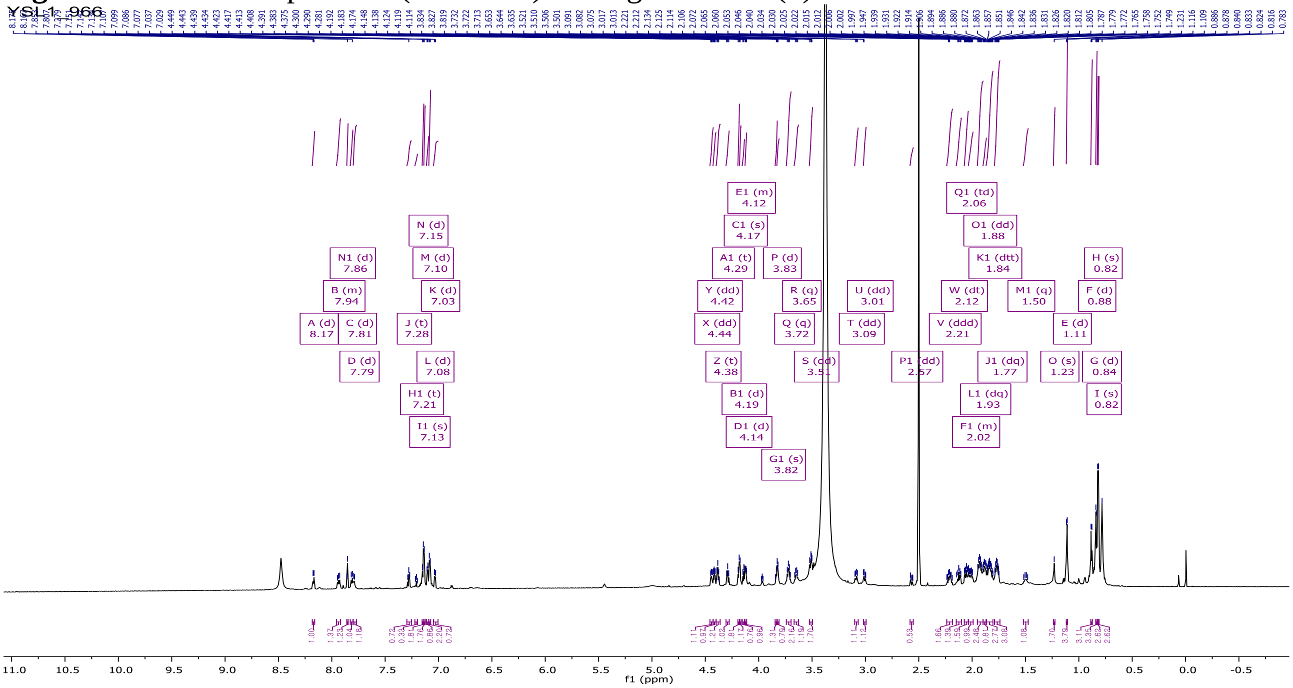

Figure S4. ^{13}C NMR spectrum (212.5 MHz) of xinghamide A (**1**) in DMSO-*d*$_6$.

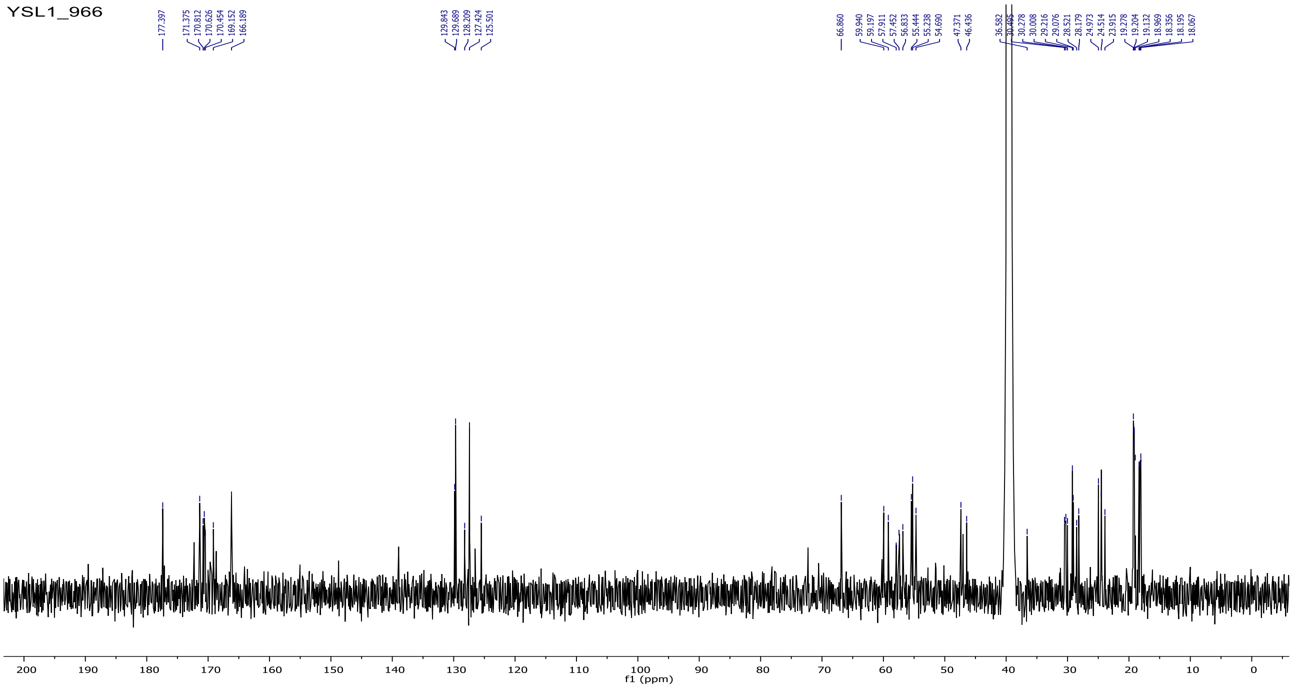

Figure S5. HSQC NMR spectrum of xinghamide A (**1**) in DMSO-*d*6.

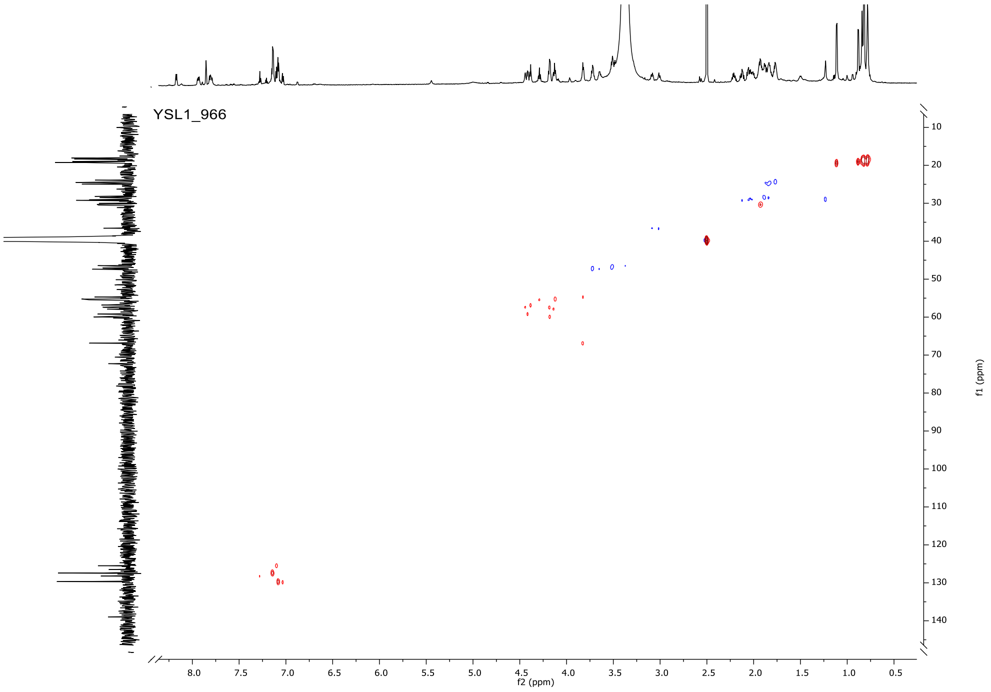

Figure S6. COSY NMR spectrum of xinghamide A (**1**) in DMSO-*d*6.

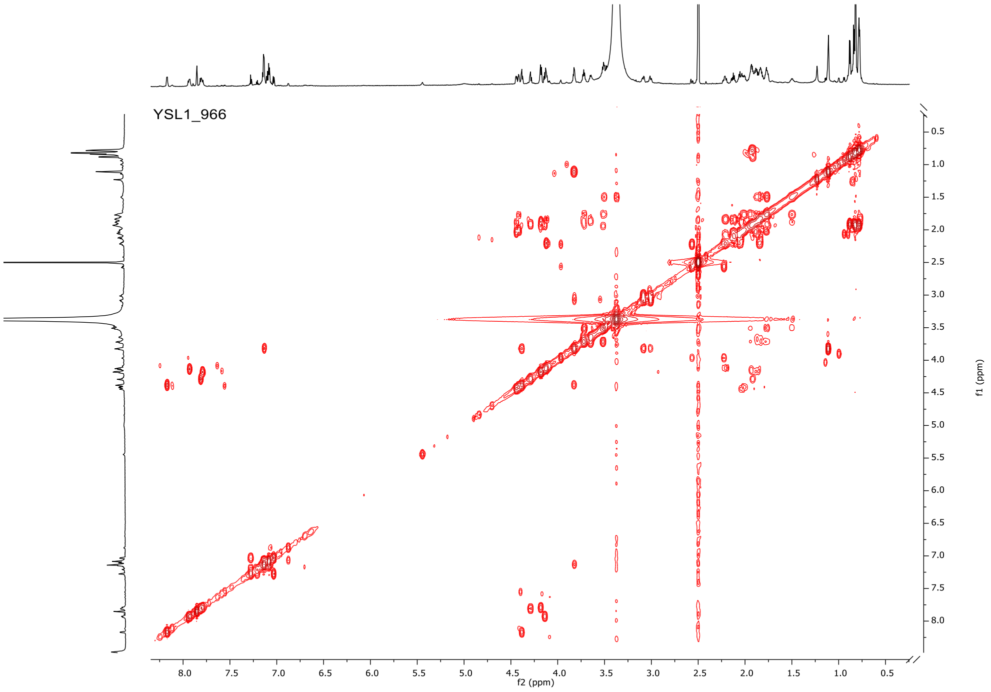

Figure S7. HMBC NMR spectrum of xinghamide A (**1**) in DMSO-d_6.

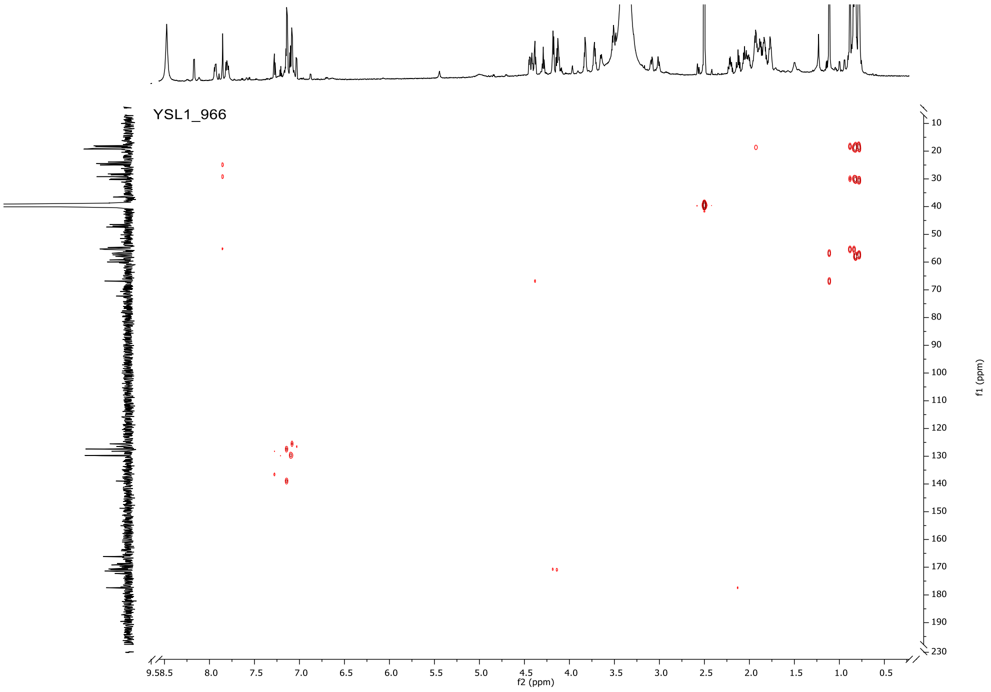

Figure S8. Magnified HMBC NMR spectrum (δ_C: 160-180 ppm; δ_H: 7.5-8.3 ppm) of xinghamide A (**1**) in DMSO-d_6.

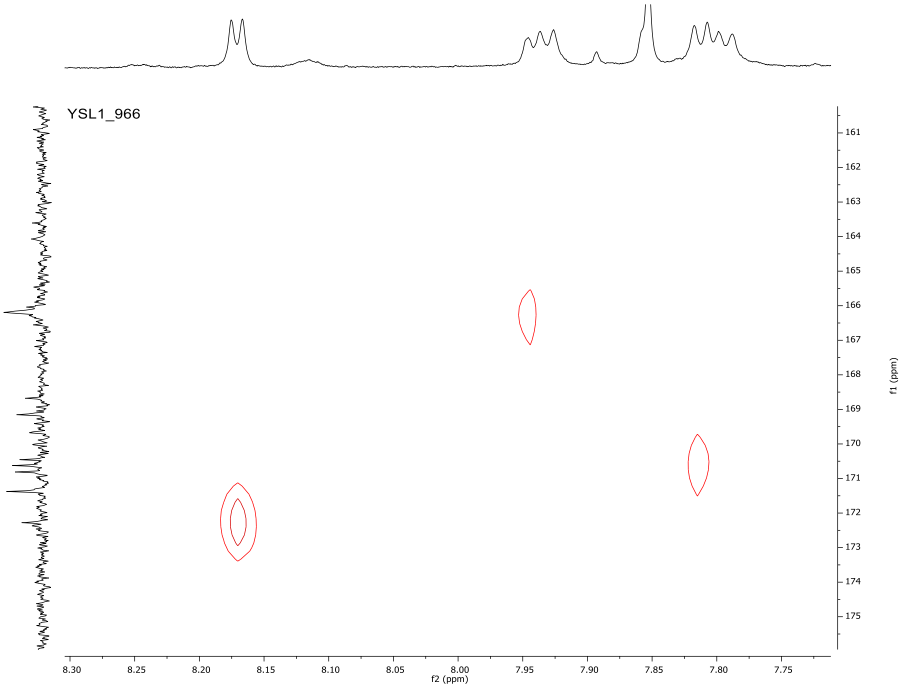

Figure S9. TOCSY NMR spectrum of xinghamide A (**1**) in DMSO-d_6.

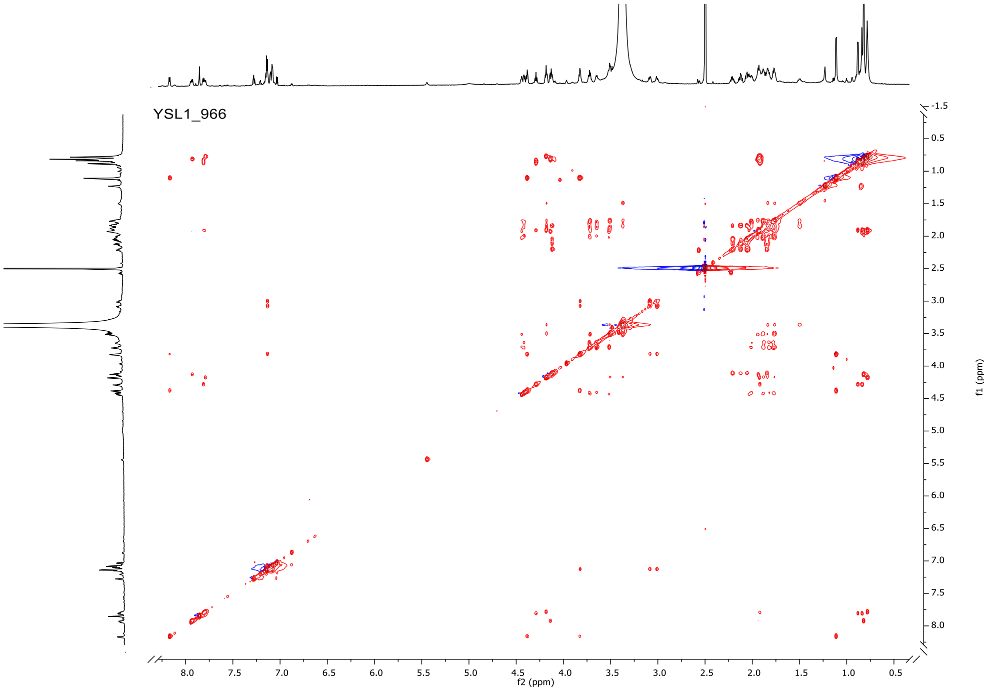

Figure S10. ROESY NMR spectrum of xinghamide A (**1**) in DMSO-d_6.

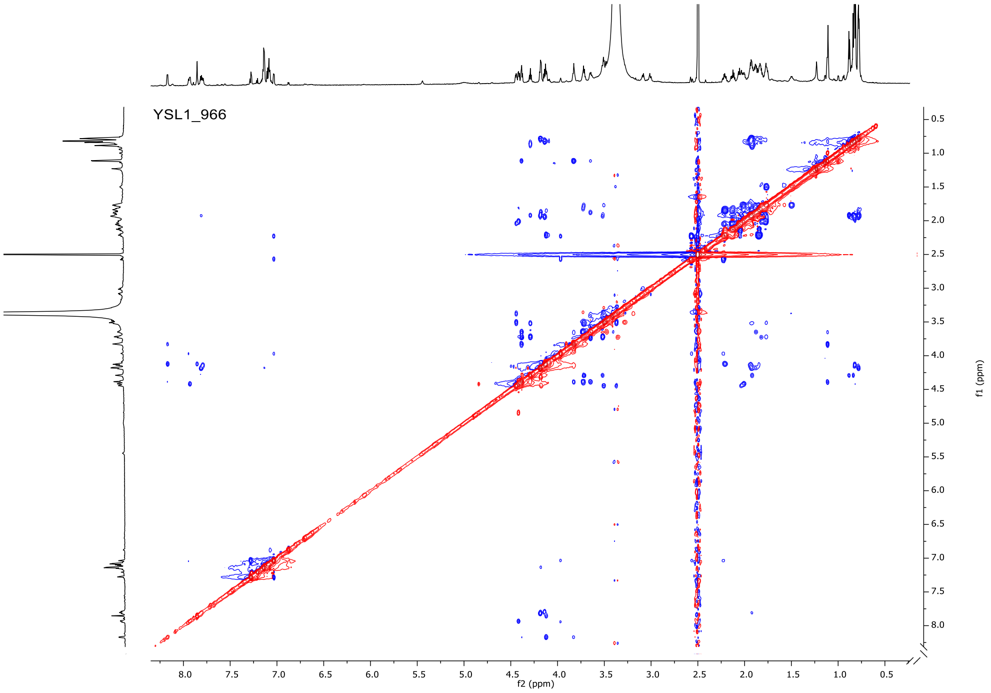

Figure S11. TIC of *S. xinghaiensis* YSL1 culture methanol crude extract and EIC of xinghamide A (**1**).

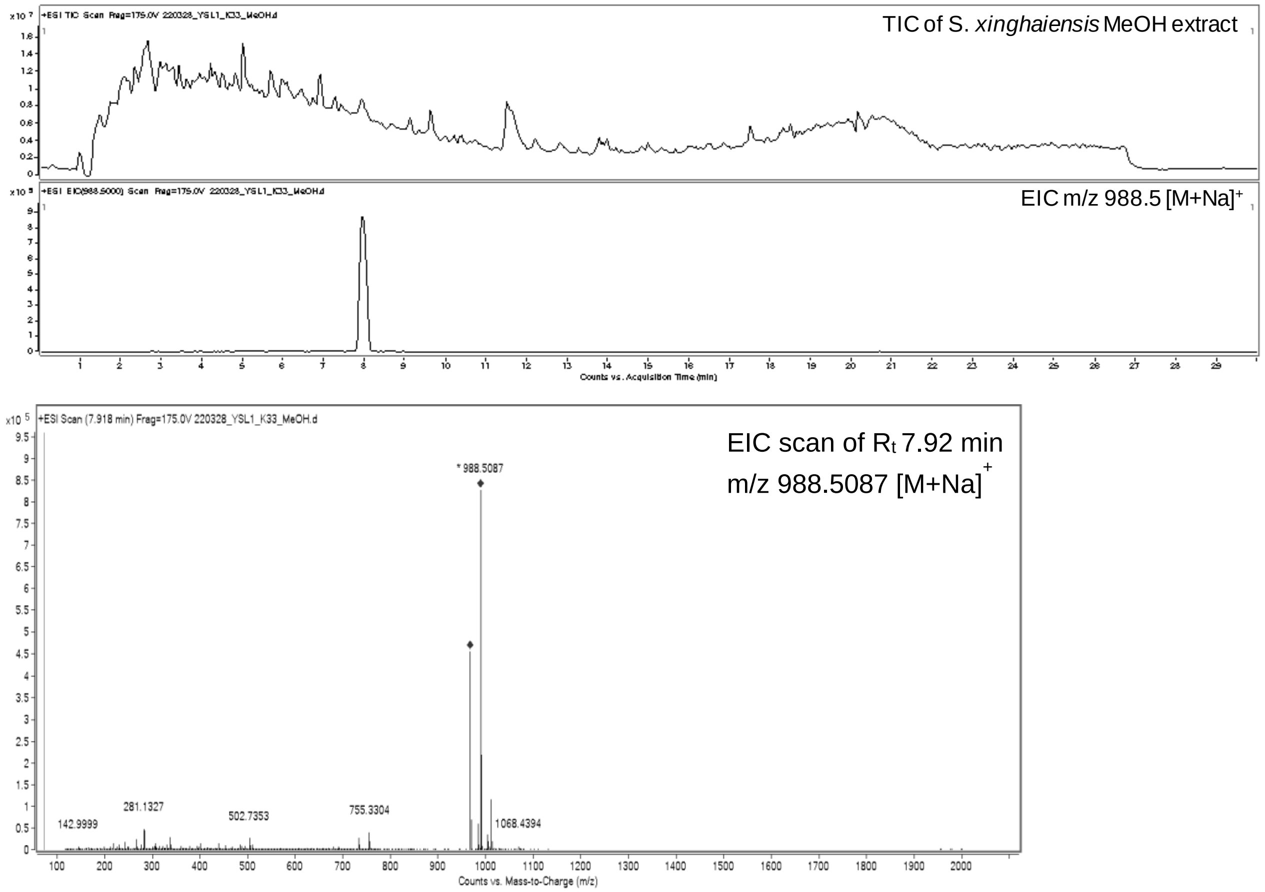

Figure S12. Advanced Marfey's analysis of xinghamide A (**1**).

(**A**) Comparison between L-FDLA and D-FDLA derivatives of threonine in **1**.

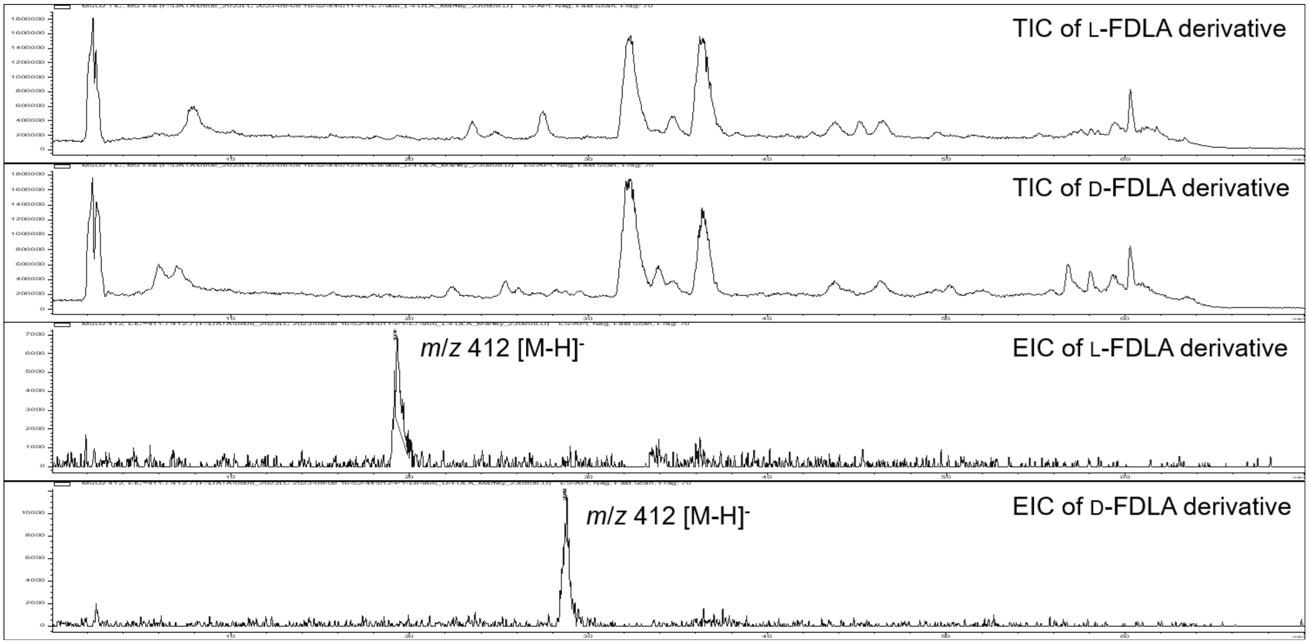

(**B**) Comparison between L-FDLA and D-FDLA derivatives of glutamic acid in **1**.

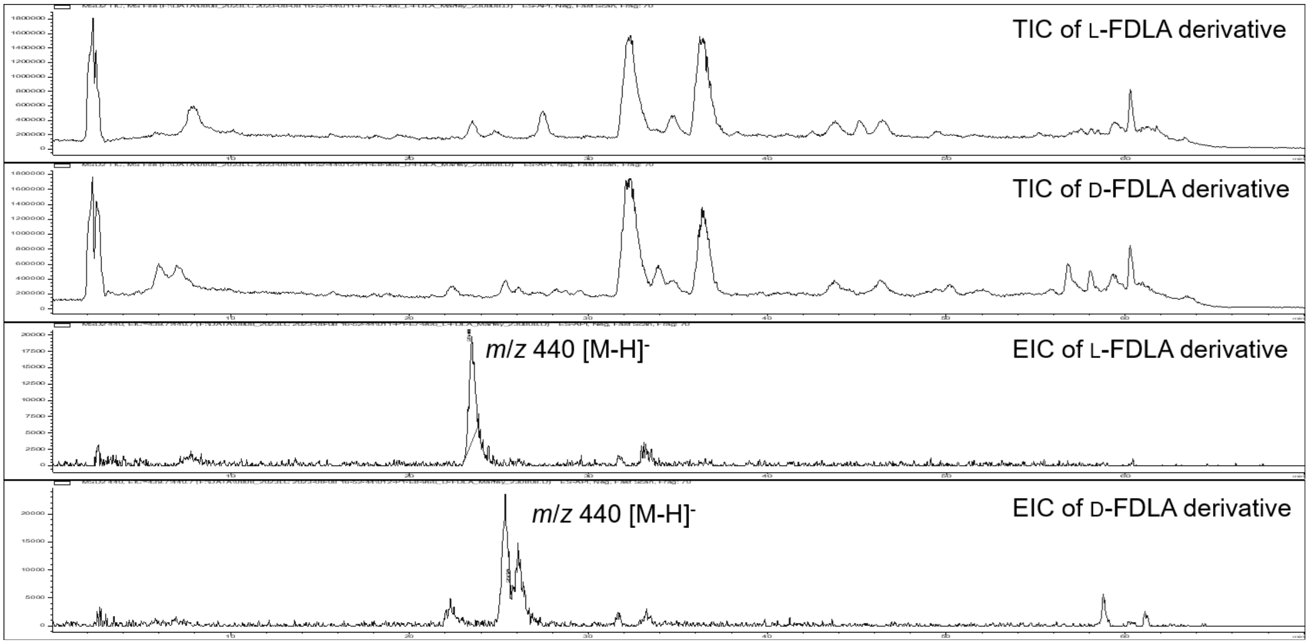

(**C**) Comparison between L-FDLA and D-FDLA derivatives of proline in **1**.

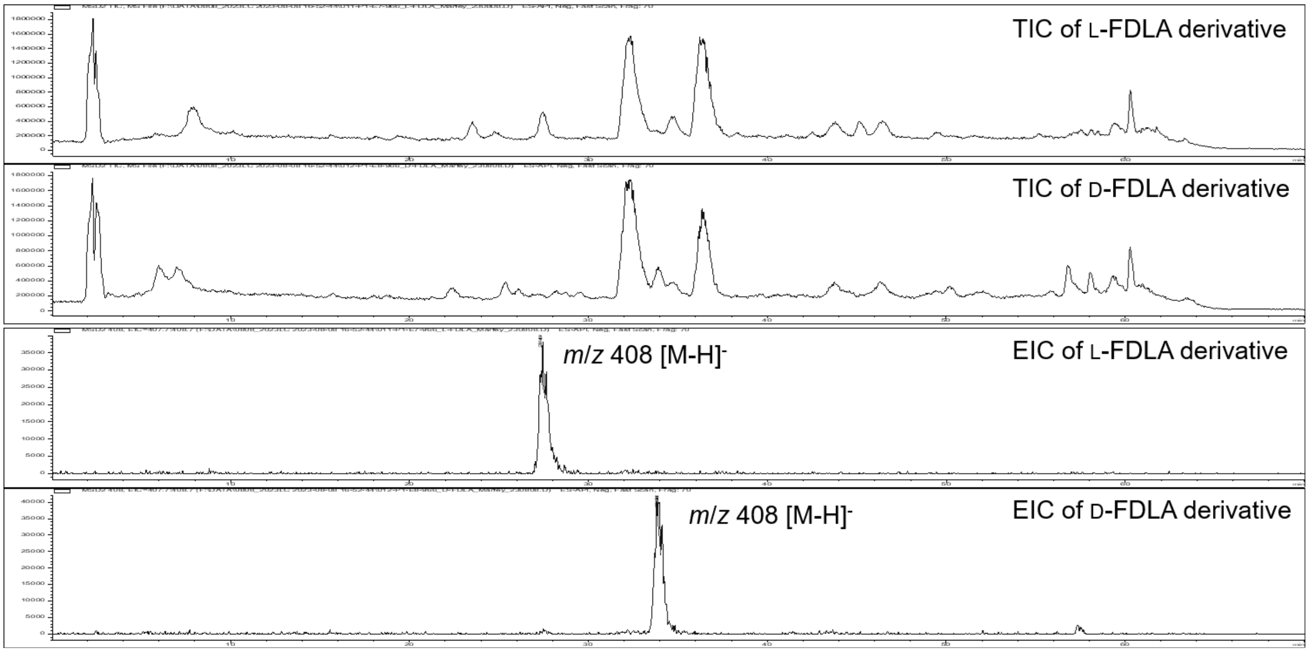

(**D**) Comparison between L-FDLA and D-FDLA derivatives of valine in **1**.

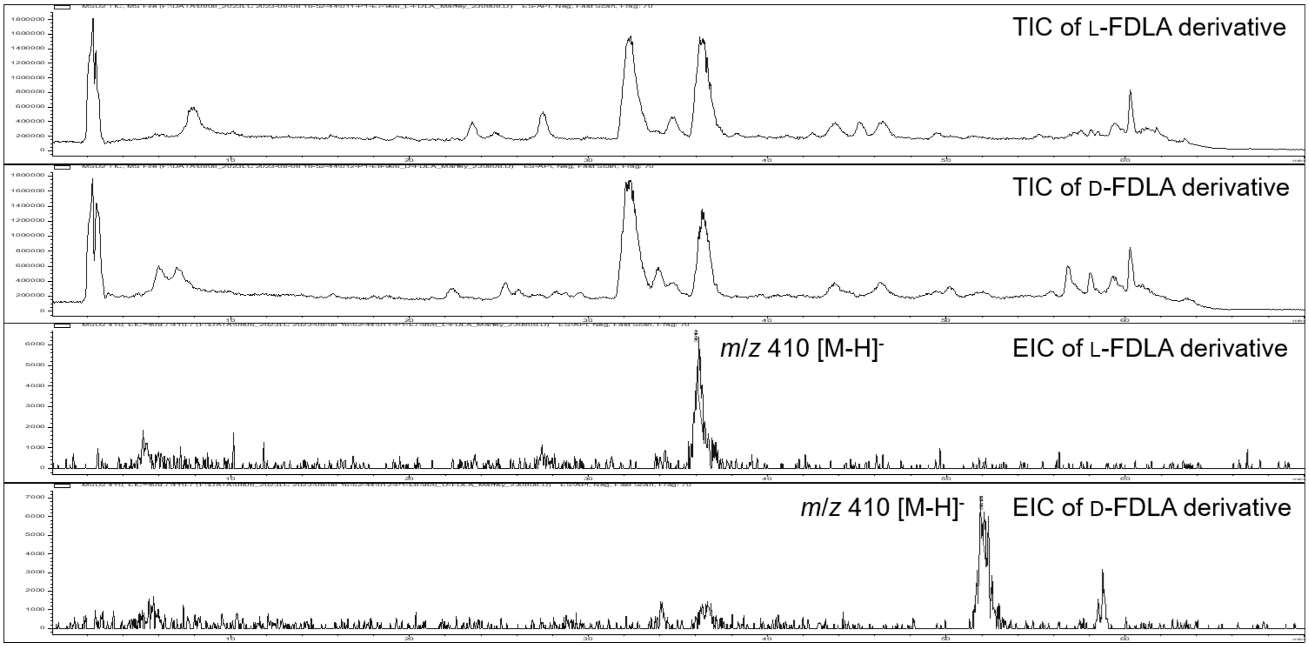

(**E**) Comparison between L-FDLA and D-FDLA derivatives of phenylalanine in **1**.

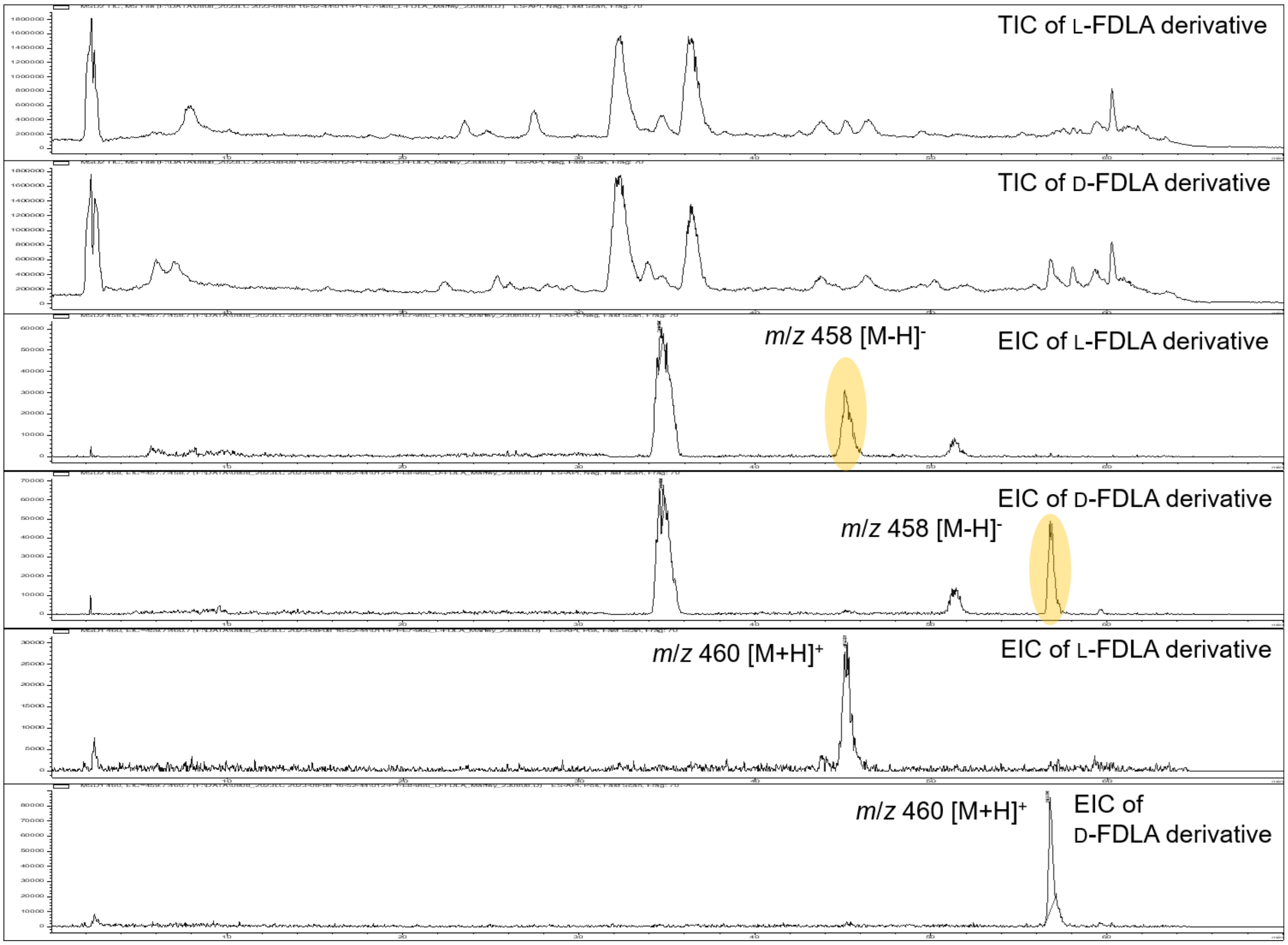

Figure S13. GITC analysis of hydrolysate of **1** to confirm configuration of β carbon of threonine in **1**.

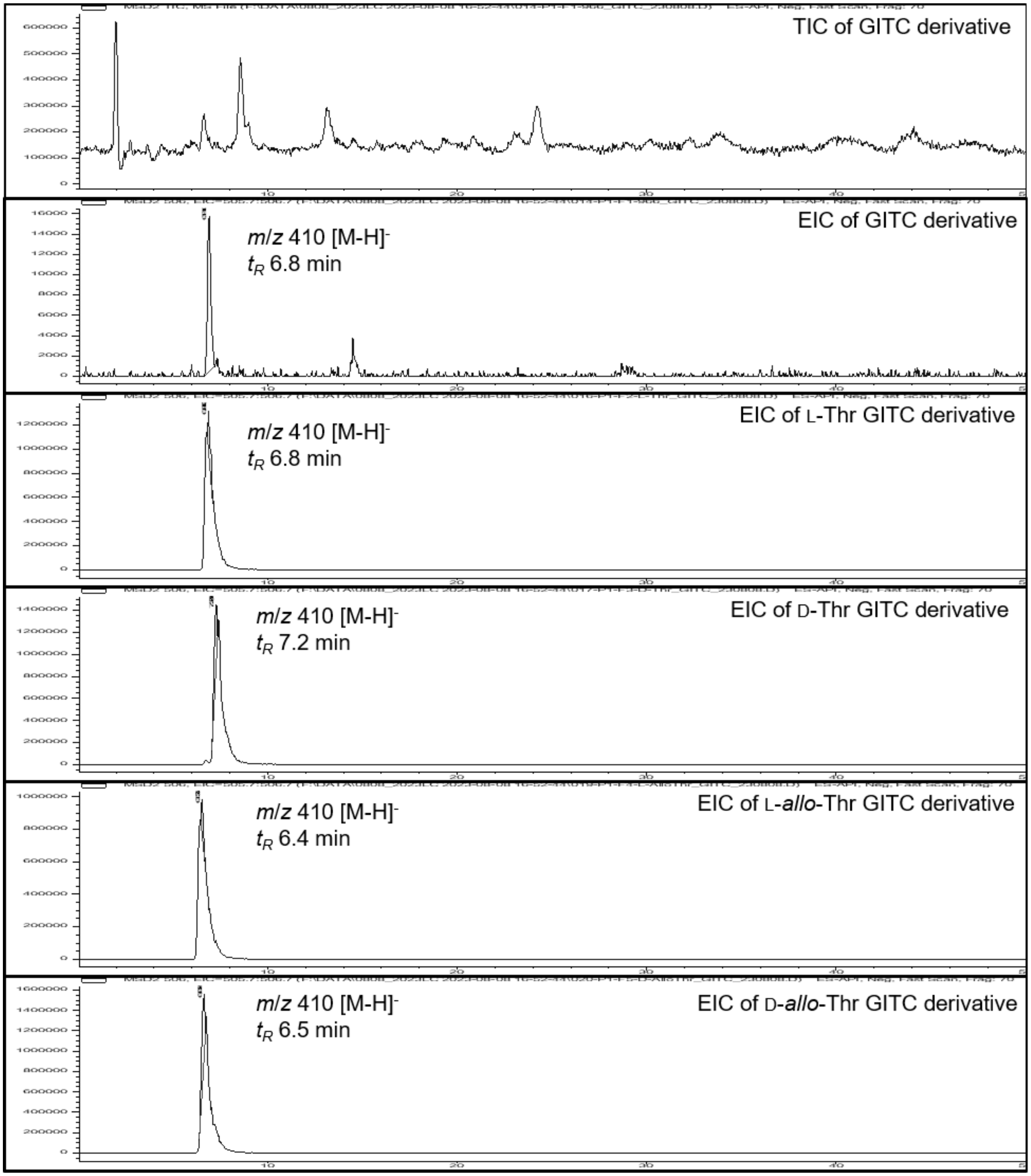

Figure S14. HRESIMS/MS analysis of xinghamide A (**1**).

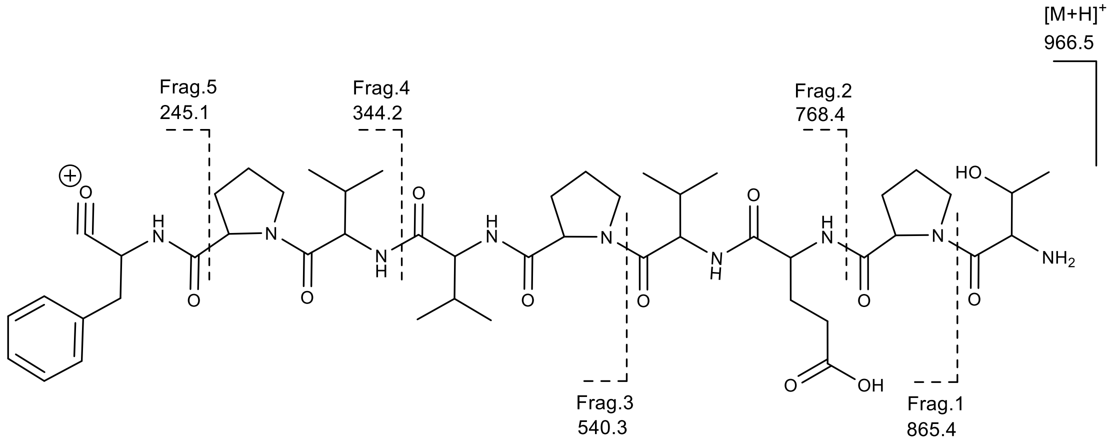

Spectrum from YSL1_966.wiff (sample 1) - YSL1_966, Experiment 1, MS^2 (100 - 2000) from 0.322 min

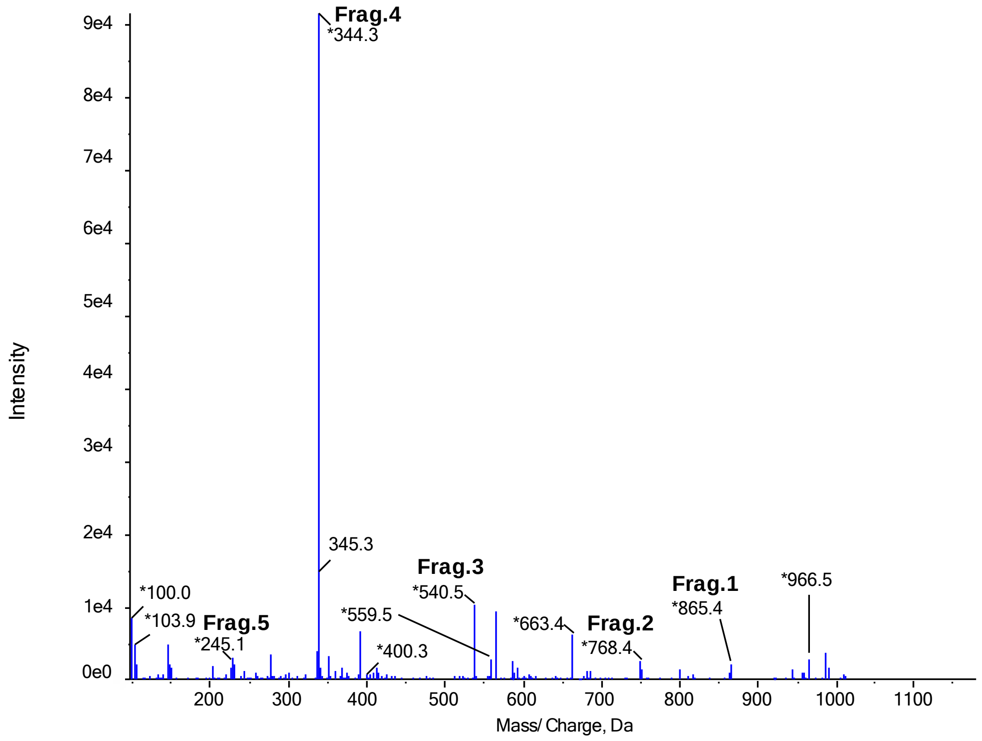

Table S1. LC/MS analysis of L, D-FDLA derivatives of the amino acids in xinghamide A (**1**).

**Amino acid**	**t_{RL} (min)**	**t_{RD} (min)**	**Elution order**	**Δt (min)**
Threonine	19.2	28.7	L→D	9.5
Glutamic acid	23.5	25.4	L→D	1.9
Proline	27.5	33.9	L→D	6.4
Valine	36.1	52.1	L→D	16
Phenylalanine	45.2	55.7	L→D	10.5
